# Potentially Inappropriate Drug Prescribing in French Nursing Home Residents: An Observational Study

**DOI:** 10.3390/pharmacy8030133

**Published:** 2020-07-30

**Authors:** Soraya Qassemi, Arnaud Pagès, Laure Rouch, Serge Bismuth, André Stillmunkes, Maryse Lapeyre-Mestre, Cécile McCambridge, Charlène Cool, Philippe Cestac

**Affiliations:** 1 Department of Pharmacy, Toulouse University Hospital, 31000 Toulouse, France; qassemi.s@chu-toulouse.fr (S.Q.); mccambridge.c@chu-toulouse.fr (C.M.); cool.c@chu-toulouse.fr (C.C.); cestac.p@chu-toulouse.fr (P.C.); 2 Department of Pharmacy, Institute of Aging, Toulouse University Hospital, 31000 Toulouse, France; rouch.l@chu-toulouse.fr; 3 Department of Primary Care, University of Toulouse, 31000 Toulouse, France; dr-bismuth@wanadoo.fr (S.B.); astillmunkes001@rss.fr (A.S.); 4 Department of Clinical Pharmacology, Toulouse University Hospital, 31000 Toulouse, France; maryse.lapeyre-mestre@univ-tlse3.fr

**Keywords:** elderly, nursing home, inappropriate prescription

## Abstract

**Purpose:** To identify the prevalence of potentially inappropriate drug prescription in a sample of nursing home residents in France, combining explicit criteria and implicit approach and to involve pharmacists in the multi-professional process of therapeutic optimization. **Methods:** A cross-sectional, observational, multicenter study was conducted during a five-month period in a sample of French nursing homes. Information on drug prescription, diseases, and socio-demographic characteristics of nursing home residents was collected. For each prescription, identification of potentially inappropriate drug prescription was done, based on explicit and implicit criteria. **Results:** Nursing home residents were administered an average of 8.1 (SD 3.2, range 0–20) drugs per day. Nearly 87% (*n* = 237) of the residents had polypharmacy with five or more drugs prescribed per day. Among the 274 nursing home residents recruited from five nursing homes, 212 (77.4%) had at least one potentially inappropriate drug prescription. According to the Laroche list, 84 residents (30.7%) had at least one drug with an unfavorable benefit–harm balance. An overdosing was found for 20.1% (*n* = 55) of the residents. Nearly 30% (*n* = 82) of the residents had a drug prescribed without valid medical indication. **Conclusions:** This study shows that potentially inappropriate drug prescriptions are highly prevalent among nursing home residents, nevertheless pharmacists can take part in drug utilization review in collaboration with the nursing home staff.

## 1. Introduction

Three types of potentially inappropriate drug prescribing (PIDP) typically exist: “overuse”, which corresponds to the prescription of more drugs than necessary; “misuse”, which corresponds to the choice of an inappropriate drug in comparison with the benefit-harm balance; “underuse”, because of an omission of treatment [1]. It was previously established that PIDP is frequent in the elderly [2]. Specifically when they live in Nursing Homes (NHs), the amount of PIDP is associated with residents, general practitioners (GPs), or NH characteristics [3]. Nursing Home Residents (NHR) with PIDP exposure also have increased risk of being hospitalized and of death [4,5]. One of the approaches used to detect potentially inappropriate drugs among the elderly is known as explicit because it is criterion-based [6] (on the establishment of drugs to be avoided or drugs to be started). These lists are generally created by expert consensus. The widely used Beers criteria [7], French Laroche list [8], the Screening Tool of Older Persons’ Prescriptions (STOPP) and the Screening Tool to Alert to Right Treatment (START) criteria are defined as explicit criteria [9,10]. Recommendations of good clinical practice provided by the French National Authority for Health (HAS), including the Clinical Practice Indicators (IPC) and Alert and Mastering of Iatrogenesis (AMI) are other examples of explicit criteria [11].

PIDP can also be detected by an implicit approach which is more random and less reproducible because it integrates a clinical judgment. Drug Utilization Review (DUR) and Medication Appropriateness Index (MAI) are part of this second approach [6,12,13].

Studies have shown interest in using these two complementary approaches to identify PIDP [14,15,16,17]. In France, to identify PIDP, systematic pharmaceutical analyses are currently implemented by pharmacists in some hospitals’ geriatrics units [18,19] but not yet in NH. These approaches are time-consuming, and pharmacists are not systematically involved to tackle this task. Additionally, the use of computer software to detect PIDP is not yet widespread [20]. By proposing therapeutic optimizations and safer drugs alternatives, the pharmacist and the NH team could reduce iatrogenic situations.

In this context, the aim of this study was to identify the prevalence of PIDP in a sample of NHR in France, combining explicit criteria and implicit approach and to assess the potential impact of a pharmacist in the multi-professional process of therapeutic optimization with nurses and general practitioners.

## 2. Materials and Methods 

### 2.1. Design

A cross-sectional, observational, and multicenter study was conducted during a five-month period. Approval for the study was granted by the management committee of each establishment and all residents gave an oral consent after information. The residents’ general practitioners were informed about the purpose of the study. The checklist items from the Strengthening the Reporting of Observational Studies in Epidemiology (STROBE) statement were used to report the study [21].

### 2.2. Data Source 

Data were collected in five public NHs around the city of Toulouse (France): Three voluntary Communal Centre of Social Action-attached NHs (CCAS-NHs) and two public Hospital-attached NHs (H-NHs). By choosing five different structures, we aimed to analyze different organizations and drug supply chains. Consent of participation was recorded automatically. Participants or guardians were informed by the physician care coordinator that their data, gathered and previously de-identified, would be used for scientific purposes. 

### 2.3. Participants 

Residents aged over 75 years and having lived in the NH for more than three consecutive months were included in the study. This minimum length of stay was chosen to focus on long-stay residents. Palliative care residents were excluded (in accordance with the physician care coordinator recommendation). 

We collected all the data from eligible NHR who were living in the CCAS-NH, but for feasibility we could not analyze all patient files from NHRs living in the H-NH—thereby we randomly selected a subset sample. Indeed, the capacity of the H-NH was much greater than the CCAS-NH, so we collected data as we went through the resident list until the end of the collection period (3 weeks).

### 2.4. Procedures

Data regarding all prescriptions in progress at the time of collection were collected by one pharmacist. Information was collected from computerized (H-NH) or not computerized (CCAS-NH) patient records. The information was transcribed into an anonymous paper file to facilitate collection on site. Then we established an Access^®^ spreadsheet (Microsoft Corporation, Redmond, Washington, DC, USA) in order to have all information on an adapted page layout and facilitate the analysis. Drugs were coded according to the anatomical therapeutic and chemical (ATC) classification system [22]. Drugs information included the non-proprietary name, forms, dosage and frequency of administration. For each prescription, identification of PIDP was done, based on explicit and implicit criteria. The explicit criteria were: (i) the French Laroche list [23], which contains drugs that should be avoided in the elderly aged over 75 years; (ii) the STOPP and START criteria [9,24,25,26]; (iii) the Summaries of the Characteristics of Products (RCP) to detect overdosing, underdosing, contraindications, drug–disease interactions and drug–drug interactions. For all prescriptions, a drug utilization review (DUR) was performed by a pharmacist, taking into account the clinical and biological context of each resident. Then, a feedback on the main results was set up in each NH by the research team. General practitioners (GP) were the main targeted audience for this feedback—they were contacted either by email or by phone to participate.

### 2.5. Outcome Measure

We used PIDP as defined in a preliminary work [17]. The primary outcome (PIDP) was coded using dichotomy (yes versus no) and was defined by the presence of at least one of the following criteria: (i) drug with an unfavorable benefit–harm balance according to the Laroche list and the residents medical conditions; (ii) drug with questionable efficacy according to the Laroche list; (iii) absolute contraindication; (iv) significant drug–drug interaction; (v) overdosing; (vi) underdosing; (vii) drug without any valid indication. We did not include the prevalence of the inappropriate drug administration (grinding of tablets or opening of capsules) in our primary outcome. However, we used the national list of “the tablets not to be crushed and the capsules not to be opened”, which was published by the French Society of Clinical Pharmacy (SFPC) to check the agreements with the methods of administration declared by the nurses [27]. 

### 2.6. Resident Characteristics

We collected demographic data (age, gender) and health status data (medical comorbidities, falls and hospitalizations during the last twelve months), as well as the consensual French version of the Mini Mental State Examination (MMSE) scores for screening for states of mental confusion [28]. Biological data were collected according to drugs prescribed (for example, the clearance of creatinine, or International Normalized Ratio (INR)). Modalities of administration related to deglutition disorders were collected among nurses. All systematic or conditional “as needed” (PRN medicines) prescriptions were recorded.

### 2.7. Nursing Home Characteristics

Information on the organization of NHs was reported by physician care coordinators or the NH director. The main structural and human resources variables were the status (public, private), and the number of medical practitioners and nurses. The main organizational variables were the presence of a pharmacy for internal usage (PIU) (yes versus no), capacities (beds), presence of a nurse at night (yes versus no), presence of computerized medical charts (yes versus no), and the existence of a special care unit for patients with dementia (yes versus no). 

### 2.8. Statistical Analysis

The qualitative variables were presented by the number and percentage of each category. Distributions of Gaussian quantitative variables were represented by the mean and standard deviation (SD). Non-Gaussian quantitative variables were expressed by their median and interquartile ranges (IQR 25th–75th percentiles). The prevalence of PIDP was obtained by bringing back the full number of residents presenting a PIDP to the full number of residents included. All analyses were performed using SAS 9.3 software (SAS Institute Inc, Cary, North Carolina, NC, USA).

## 3. Results

### 3.1. Characteristics of NHs and Study Population (Residents)

The number of general practitioners intervening in the NH varied from 1 to 25 per hundred beds. All NHs were public, located in urban area and are entitled to accept social assistance. The NHs structural and organizational characteristics are presented in Table 1.

**Figure 1:**
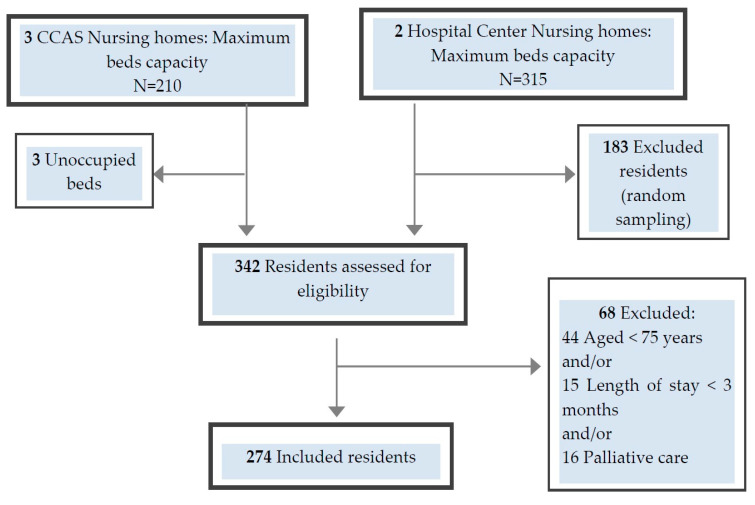
Flowchart of residents included in the study. Legend: CCAS, Communal Centre of Social Action.

A flow chart is presented in Figure 1. 

Residents’ main characteristics are presented in Table 2. The mean age was 88.9 (SD 6.2, range 75–105). The most common comorbidities were hypertension (64.6%), dementia (53.3%), and depression (47.5%).

### 3.2. Characteristics of Drugs Prescription

An average of 8.1 (SD 3.2, range 0–20) drugs per day and per resident was found. Nearly 87% (*n* = 237) of the residents had polypharmacy with five or more drugs prescribed per day (Table 3).

### 3.3. Outcomes Measures 

Among the 274 residents, 212 (77.4%) had at least one PIDP. The descriptions of the drug-related problems are presented in Table 4.

According to the Laroche list, 84 residents (30.7%) had at least one drug with an unfavorable benefit–harm balance and 6 (2.2%) had at least one drug with questionable efficacy. In total, 7.3% residents (*n* = 20) presented at least an absolute contraindication, and 2.6% (*n* = 7) a major drug interaction. An underdosing and overdosing was found, respectively, for 4% (*n* = 11) and 20.1% (*n* = 55) of the residents. Nearly 30% (*n* = 82) of the residents had a drug prescribed without valid medical indication, and 27% residents (*n* = 74) had duplication of drugs belonging to the same therapeutic class. In total, 15.3% (*n* = 42) of the residents had a partially treated indication for which a synergistic drug or corrector had to be associated and 7.3% (*n* = 20) of the residents had no effective treatment for a condition for which one or several drug classes demonstrated their efficacy. Table A1, Table A2, Table A3, Table A4 and Table A5 (in Appendix A) presents the most frequent inappropriate drugs prescribed. 

During the feedback planned in each NH, the research team presented the main results and heightened awareness measures among the staff. The physicians care coordinators, the middle managers, and the director were always present. Some GPs, nursing staff (nurses, nurse’s aides), pharmacists and family representatives also took part at some meetings. 

## 4. Discussion

The aim of our study was to identify PIDP among French nursing home residents. Our results show that the PIDP prevalence reached 77.4% using both implicit and explicit approaches. 

The prevalence of PIDP is comparable with studies conducted in European NHs. The prevalence varies from 20 to 79% depending on the countries and the explicit criteria used [29]. However, it is well known that PIDP prevalence is criteria-dependent [30,31], therefore it seems difficult to compare our results with previous studies since we used a broader definition of PIDP, integrating a set of heterogeneous criteria.

If we focus on the Laroche list, we found that 32.9% of the residents had a drug with an unfavorable benefit–harm balance or a questionable efficacy. In France, Bongue and al. carried out the first national evaluation of PIDP prevalence [32]. It was based on the Laroche list from data of the health insurance and it found a PIDP prevalence of 53.6% in the elderly aged 75 years or older. Our results are similar to those of Cool and al. [17], who recently carried out a study in French NHs. They found nearly 71% of PIDP among 974 NHRs by using a similar methodology. 

The median number of drugs prescribed per day is similar to those found in the literature [33]. Regarding the polypharmacy, 118 residents (43.1%) concurrently used 5 to 8 drugs. These results are consistent with a recent study which found 49.7% of polypharmacy (5–9 drugs) among European NHR (*n* = 4156) [34]. In our sample, only 37 residents (13.5%) had less than five drugs and were not polymedicated. However, an association between inappropriate prescribing and polypharmacy is well defined [2]. Compare to our results, Onder and al. showed that laxatives, antiacid and antiplatelet drugs were the most prescribed drugs in European NHRs [34]. 

Regarding the drugs with an unfavorable benefit–harm balance according to the Laroche list, our results showed that the most prescribed potentially inappropriate drug classes were benzodiazepine-related drugs and derivatives, and atropinic drugs (Table A1, Table A2, Table A3, Table A4 and Table A5 in Appendix A). These results are confirmed by many studies which found the same principal prescribed potentially inappropriate drug classes [32,35]. Regarding the pharmacological redundancy, 33 residents (12%) were prescribed three psychotropic drugs or more. A significant proportion of psychotropic drugs prescribed were full dose hypnotics (zolpidem > 5 mg/day, zopiclone > 3.5 mg/day, lormetazepam > 0.5 mg/day). These drugs belong to the Laroche list and are known to have potentially serious side effects [36]. However, psychotropics are widely used in nursing home settings. Several reasons can explain this situation. First, the NH medical staff is reduced during the night [37]. Second, there is a high prevalence of residents with dementia, which may cause communication issues with poorly trained medical staff. This hypothesis is confirmed by the fact that several successful interventions with teams better trained to deal with behavioral problems have a positive impact on psychotropic drug prescriptions [38,39]. Finally, residents go to bed earlier in these institutions and and thus need high doses of hypnotics to deal with night awakenings. 

One of the most frequent PIDPs was the overprescribing of drugs without any valid indication. According to previous studies, we found that proton pump inhibitors (PPIs) are the most involved overprescribing drugs among the elderly [40,41]. The simultaneous use of an antithrombotic or the absence of treatment revaluation can explain this overprescribing [42]. Not only the fact that PPIs were widely prescribed without indication, they were also overdosing when we found a valid indication (prevention of ulcers or prevention of gastrointestinal bleeding in patients on antithrombotic treatments) [43]. A double dose of pantoprazole, esomeprazole or lansoprazole was found in 13 residents (4.7%) instead of a standard dose. In many cases, the maintenance dose is to take half of the full dose once a day. Different studies have highlighted an increased rate of adverse reaction (decrease vitamin and mineral absorption, osteoporotic-related fractures, pneumonia, *Clostridium difficile* infection) with long-term PPIs use [44] and overdosing. 

Agents acting on the renin–angiotensin system were under-prescribed in our sample, whereas angiotensin II receptor blockers and angiotensin-converting enzyme inhibitors are effective to reduce all-cause mortality and cardiovascular mortality in people at high risk of heart failure [45]. A lot of scientific reports converged towards the same conclusion, namely that they represent the pillar of the treatment of heart failure [46].

The overdosing of antithrombotic agents was defined in our study by a dose of acetylsalicylic acid higher than 100 mg/day. In total, 13 residents (4.7%) had 160 mg per day of acetylsalicylic acid despite the fact that studies showed that a higher dose offered no additional benefits (300 mg vs. 50–100 mg/day) [47]. Furthermore, these drugs tend to be overprescribed, and no valid indication was found for 18 residents (6.6%). Studies notes that acetylsalicylic acid is still overprescribed for stroke prevention in atrial fibrillation even though it has no efficacy in this indication [48,49]. We must also point out the fact that the transient ischemic attacks were not thoroughly recorded in the residents’ medical file.

Finally, 45 residents (16.4%) had inadequate administration (crushing tablets or opening of capsules) and nervous system drugs were often crushed. Deglutition disorders and agitation states can explain this practice [50]. Nevertheless, these manipulations are not insignificant as far as active substances are concerned, because they can lower therapeutic effectiveness and could even be toxic. This kind of process can involve a loss of chance for the patients as well as a considerable economic loss. In addition, nurses responsible for crushing consequently incur several chemical and biological risks (powder inhalation). 

Nurses and nurses’ aides should systematically contact the pharmacist and the GP when facing difficulties related to administration or doubts about the presence of tolerance problems. The detection of PIDP would thus be facilitated with more interaction and reciprocal recognition.

Some limitations of the present study need to be recognized. First, we recorded all conditional prescriptions and some data may be missing from medical records. This could have determined an overestimation of the overall PIDP prevalence and some prescriptions may have been justified if the prescriber had been interviewed. Second, we used heterogeneous criteria to define our principal outcome. Therefore, it may be difficult to compare our results with those of other studies because our method may overestimate the PIDP rate, since in most studies the outcome is defined by only one or two criteria. However, it is possible to compare the rate of drug prescriptions with unfavorable benefit–harm balance belonging to explicit criteria (Laroche list). 

During the feedback of the main results we identified several obstacles—for instance, the inability to bring together all the participants involved. We were faced with the weak mobilization of the GPs and the lack of dedicated time for the nurses. Indeed, the first ones cannot easily leave their practice without financial compensation, and the later are under-staffed within the structure and therefore cannot find sufficient time. These problems will be taken into account for later studies. Finally, it should be underlined that the weak mobilization of the GP was a barrier to therapeutic optimization and having a General Practitioner–Pharmacist collaboration would have allowed better and more detailed prescription analysis, as mentioned by Bryant and al. [51]. 

In spite of this, our study also has strengths. Data were collected by the same health professionals throughout the whole study. We combined explicit and implicit criteria to optimize pharmaceutical analysis, as well inadequate administration practices. We also took the time to heighten awareness measures among the NH staff during the restitutions of the results so it may improve their medical practice and thereby spread our methodology for other residents.

## 5. Conclusions

In conclusion, this study shows that PIDPs are highly prevalent among NHRs, nevertheless pharmacists and nurses can take part in drug utilization reviews in collaboration with the nursing home staff, and by being active members of a multidisciplinary team. This study had positive feedback and encouraged more research on the subject. Therefore, we intend to schedule, in the near future, an intervention program designed to improve prescribing practices and communication tools with GPs. Since this study, community pharmacists in France have been able to achieve medication review among polymedicated NHR and they are paid for this task. This could facilitate the implementation of pharmaceutical analysis in NH. 

## Figures and Tables

**Table 1:** Nursing home main characteristics (*n* = 5).

Nursing Home Characteristics	CCAS-NH			H-NH	
	1	2	3	1	2
Pharmacy for internal usage	No (community pharmacist)	No (community pharmacist)	No (community pharmacist)	Yes	Yes
Maximum capacities (beds)	60	65	85	195	120
Special care unit	Yes	No	Yes	Yes	No
Presence of a nurse at night	No	No	Yes	Yes	No
Computerized medical charts	No	No	No	Yes	Yes
GP per 100 beds	25	18.5	8.2	1	19.2
Nurses per 100 beds	6.7	6.1	8.2	Δ	7.5

Δ Missing data: data collection was not applicable because there were a lot of temporary workers. Abbreviations: General Practitioner (GP); Nursing Home of the Communal Centre of Social Action (CCAS-NH); Nursing Home attached to a public hospital (H-NH).

**Table 2:** Residents’ main characteristics (*n* = 274).

Resident Main Characteristics	Total *n* = 274	%	Mean	SD	Median	p25%	p75%
**Demographic**							
**Age** (year)			88.9	6.2			
**Gender**							
Men	67	24.5					
**Dementia**							
**MMSE score** ^Δ^					15	9	21
**Biological data**							
**Renal function** ^Δ^ **(Cockcroft–Gault formula)**			44.2	18.3			
**Hospitalization in the previous 12 months** ^Δ^					0	0	1
**Falls in the last 12 months** ^Δ^					1	0	3
**Most common comorbidities**							
Hypertension	177	64.6					
Dementia (Alzheimer’s disease and other)	146	53.3					
Depression	130	47.5					
Arthrosis	62	22.6					
Atrial fibrillation	61	22.3					
Hypothyroidism	55	20.1					
Diabetes	54	19.7					
Osteoporosis	50	18.3					
Psychosis, excluding depression	43	15.7					
Stroke	42	15.3					
Myocardial infarction	37	13.5					
Congestive heart failure	36	13.1					
Hypercholesterolemia	34	12.4					
Deep-vein thrombosis	30	11					

MMSE: Mini Mental States Examination. Δ Missing data: *n* = 61 for MMSE score. *n* = 46 for renal function. *n* = 2 for falls and hospitalizations.

**Table 3:** Main characteristics of drugs prescription.

Drug	Total *n* = 274	%
**No polypharmacy (0–5 drugs)**	61	22.3
**Polypharmacy (6–8 drugs)**	94	34.3
**Hyperpolypharmacy (≥9 drugs)**	119	43.4
**Number of patients with a least one prescription of the anatomical class**		
**N** Nervous System	255	93.1
**A** Alimentary tract and metabolism	241	88
**C** Cardiovascular System	201	73.4
**B** Blood and blood forming organs	146	53.3
**H** Systemic hormones, excluding sex hormones	60	21.9
**Therapeutic class**		
**N05** Psycholeptics	204	74.5
**N02** Analgesics	173	63.1
**A06** Drugs for constipation	139	50.7
**N06** Psychoanaleptics	137	50
**B01** Antithrombotic Agents	132	48.2

**Table 4:** Prevalence and description of potentially inappropriate prescribing (PIDP) among residents (*n* = 274).

Potential Drug-Related Problems	Residents *n* = 274	%
**Primary outcome measure (at least one PIDP)**	212	77.4
**Non-compliance to the various consensus**		
Drug(s) with a non-favorable benefit-to-risk ratio		
According to the Laroche list	84	30.7
According to clinical and biological residents’ data	38	13.9
Presence of drug(s) with questionable efficacy		
According to the Laroche list	34	12.4
**Absolute contraindication**	20	7.3
**Significant drug–drug interaction**	7	2.6
**Underdosing**	11	4
**Overdosing**	55	20.1
**Concomitant prescription of drugs from the same therapeutic class**	74	27
Concomitant prescription of psychotropic drugs ≥3 † :	33	12
≥2 antipsychotics †	7	2.6
≥2 benzodiazepines †	11	4
≥2 antidepressants †	1	0.4
Concomitant prescription of diuretics: ≥2 †	4	1.5
Concomitant prescription of antihypertensive drugs: ≥4 †	7	2.6
Other redundancies	11	4
**Drugs without any valid indication**	82	30
**Under-prescribing**	62	22.6
Absence of an effective treatment for a condition for which one or several drug classes have demonstrated their efficacy	20	7.3
Absence of a synergistic drug or corrector	42	15.3

† The Clinical Practice Indicators (IPC), Alert and Mastering of Iatrogenesis (AMI) from the French Health Authority.

## Appendix A

**Table A1:** Most frequent inappropriate drugs prescribed according to each component of PIDP (*n* = 274). (Part 1).

Components of PIDP	Drug Class	Non-Proprietary Name	Residents. No. (%)
**Drug with an unfavorable benefit-to-risk ratio according to the Laroche list**			
	Hypnotics and sedatives	Zolpidem ^a^	16 (5.9)
		Zopiclone ^b^	16 (5.9)
		Lormetazepam ^c^	7 (2.6)
		Clorazepate	2 (0.7)
	Anxiolytics	Bromazepam	8 (3)
		Oxazepam ^d^	8 (3)
		Hydroxyzine	7 (2.6)
		Diazepam	4 (1.5)
		Prazepam	3 (1.1)
		Levomepromazine	1 (0.4)
	Antihistaminics	Alimemazine	8 (3)
		Oxomemazine	2 (0.7)
	Antipsychotics	Cyamemazine	5 (1.8)
**Drug with questionable efficacy, according to the Laroche list**			
	Non-steroids anti-inflammatory and antirheumatic products	Diclofenac	11 (4)
		Niflumic acid	5 (1.8)
		Chondroitin sulfate	1 (0.4)
	Anti-dementia drugs	Ginkgo folium §	4 (1.5)
	Other anxiolytics	Etifoxine	4 (1.5)
	Antivertigo	Betahistine ^e^	3 (1.1)
		Acetylleucine	2 (0.7)
	Peripheral vasodilators	Naftidrofuryl §	2 (0.7)
	Other cardiac preparations	Trimetazidine	1 (0.4)

^a^ Not recommended for dose greater than 5 mg/day. ^b^ Not recommended for dose greater than 3.75 mg/day. ^c^ Not recommended for dose greater than 0.5 mg/day. ^d^ Not recommended for dose greater than 60 mg/day. ^e^ Never studied in elderly and moderate efficacy. § Drugs belonging to the 2007 Laroche list.

**Table A2:** Most frequent inappropriate drugs prescribed according to each component of PIDP (*n* = 274). (Part 2).

Components of PIDP	Drug Class	Non-Proprietary Name	Residents. No. (%)
**Other Drug with an unfavorable benefit-to-risk ratio**			
Elderly	Cardiovascular system	Nitroglycerine	8 (2.9)
		Verapamil	4 (1.5)
		Ditilazem	3 (1.1)
		Tropatepine	1 (0.4)
	Nervous system	Paracetamol + opium + caffeine	3 (1.1)
		Tropatepine	1 (0.4)
	Musculo–skeletal system	Allopurinol	6 (2.2)
		Colchicine and opium association	1 (0.4)
No psychiatric disease, no aggressivity	Nervous system	Risperidone	3 (1.1)
		Haloperidol	1 (0.4)
Diabetes	Nervous system	Tramadol, paracetamol	3 (1.1)
Dementia	Genito urinary system and sex hormones	Trospium	1 (0.4)
Hypertension, heart failure	Nervous system	Paracetamol effervescent	1 (0.4)
Off-label prescription	Nervous system	Clonazepam	1 (0.4)

**Table A3:** Most frequent inappropriate drugs prescribed according to each component of PIDP (*n* = 274). (Part 3).

Components of PIDP	Non-Proprietary Name	Residents. No. (%)
**Drug–disease contraindication**		
Severe renal impairment	Irbesartan, hydrochlorothiazide	1 (0.4)
	Spironolactone	1 (0.4)
	Alendronique acid	1 (0.4)
	Hydroclorothiazide	1 (0.4)
	Diclofenac	1 (0.4)
Hyperkalemia	Spironolactone	2 (0.7)
Glaucoma	Oxomemazine	1 (0.4)
	Solifenacine	1 (0.4)
	Hydroxyzine	1 (0.4)
Active zoster	Prednisone	1 (0.4)
Active depression	Rilmenidine	1 (0.4)
Medical history of stroke	Tuaminoheptane	1 (0.4)
Asthma	Codeine, chlorhydrate ethylmorphine	1 (0.4)
Iron overload	Sodium feredetate	1 (0.4)
Elderly	Fosfomycine	1 (0.4)

**Table A4:** Most frequent inappropriate drugs prescribed according to each component of PIDP (*n* = 274). (Part 4).

Components of PIDP	Drug Class	Non-Proprietary Name	Residents. No. (%)
**Drug–drug contraindication**			
Drugs which prolongs the QT interval		Citalopram with Domperidone	2 (0.7)
		Citalopram with Haloperidol	1 (0.4)
		Escitalopram with Sotalol	1 (0.4)
		Escitalopram with Haloperidol	1 (0.4)
		Escitalopram with Domperidone	1 (0.4)
		Flecaine with Bisoprolol	1 (0.4)
**Significant drug–drug interaction**			
Increase in hemorrhagic risk		Acetyl-salicylique acid with Clopidogrel ^f^	1 (0.4)
Reciprocal antagonism		Haloperidol with Levo-DOPA	1 (0.4)
		Cyamemazine with Levo-DOPA	1 (0.4)
Association not recommended, increased risk of ventricular arrhythmia		Haloperidol with Cyamemazine	1 (0.4)
		Haloperidol with Levomepromazine	1 (0.4)
Disorder of the cardiac conduction		Diltiazem with Carteolol	1 (0.4)
**Absence of an effective treatment for a valid indication: an effective therapeutic had to be associated**			
Osteoporosis ^g^		Vitamin D	12 (4.4)
Atrial Fibrillation	Antithrombotic agents (vitamin K antagonists or platelet aggregation inhibitors)		5 (1.8)
Secondary cardiovascular prevention post-Myocardial infarction	Agents acting on the renin–angiotensin system and platelet aggregation inhibitors ± betablockers		3 (1.1)
Secondary cardiovascular prevention post stroke	Platelet aggregation inhibitors		2 (0.7)
	Platelet aggregation inhibitors and HMG CoA reductase inhibitors ^h^		1 (0.4)

^f^ No indication found at a dual anti-platelet aggregation (acute coronary syndromes). ^g^ Criteria not included in our results because an administration of vitamin D may have occurred before the study. ^h^ We recommended replacement with a statin for secondary prevention (simvastatin, pravastatin, fluvastatin) only if a statin for primary prevention is prescribed [52]. If no statin is prescribed, we do not propose to introduce any statin.

**Table A5:** Most frequent inappropriate drugs prescribed according to each component of PIDP (*n* = 274). (Part 5).

Components of PIDP	Drug Class	Residents. No. (%)
**Indication partially treated: it was necessary to add a synergistic drug or corrector**		
Heart failure	Agents acting on the renin–angiotensin system or Betablockers	13 (4.7)
	Agents acting on the renin–angiotensin system and/or diuretics	3 (1.1)
	Betablockers or diuretics	2 (0.7)
Prevention opioid-induced constipation	Laxatives drug	8 (2.9)
Secondary cardiovascular prevention post Myocardial infarction	HMG CoA reductase inhibitors ^h^	2 (0.7)
	Platelet aggregation inhibitors	1 (0.4)
Chronic obstructive arterial disease	Platelet aggregation inhibitors	1 (0.4)
**Indication partially treated: it was necessary to add a synergistic drug or corrector**		
Heart failure	Agents acting on the renin–angiotensin system or Betablockers	13 (4.7)
	Agents acting on the renin–angiotensin system and/or diuretics	3 (1.1)
	Betablockers or diuretics	2 (0.7)
**Prescribing without valid medical indication**		
	Proton pump inhibitors	28 (10.2)
	Antithrombotic Agents	18 (6.6)
	Diuretics	12 (4.4)
	Anti-dementia drugs	9 (3.3)
	Drugs for obstructive airway diseases (adrenergics and others)	5 (1.8)
	Antiarrythmics (class I and III)	4 (1.5)
	Anti-epileptics	4 (1.5)
	Betablockers	3 (1.1)
	Calcium channel blockers	3 (1.1)
	Antihistamines for systemic use	3 (1.1)

^h^ We recommended replacement with a statin for secondary prevention (simvastatin, pravastatin, fluvastatin) only if a statin for primary prevention is prescribed [52]. If no statin is prescribed, we do not propose to introduce any statin. Abbreviation: HMG CoA: hydroxymethylglutaryl Coenzym A reductase.
